# Older adult health and primary care: autonomy, vulnerabilities and challenges of care

**DOI:** 10.11606/s1518-8787.2021055002856

**Published:** 2021-05-06

**Authors:** Jeane Lima e Silva Carneiro, José Ricardo de Carvalho Mesquita Ayres

**Affiliations:** I Universidade São Paulo Faculdade de Medicina da Universidade de São Paulo Programa de Pós-Graduação em Saúde Coletiva São PauloSP Brasil Universidade São Paulo. Faculdade de Medicina da Universidade de São Paulo. Programa de Pós-Graduação em Saúde Coletiva, São Paulo, SP, Brasil; II Universidade de São Paulo Faculdade de Medicina da Universidade de São Paulo Departamento de Medicina Preventiva São PauloSP Brasil Universidade de São Paulo. Faculdade de Medicina da Universidade de São Paulo. Departamento de Medicina Preventiva, São Paulo, SP, Brasil

**Keywords:** Older Adult, Personal Autonomy, Health Vulnerability, Health-Disease Process, Aging, Primary Health Care

## Abstract

**OBJECTIVE:**

To understand the relations between autonomy and health-disease-care processes of older adults in the daily life of primary health care.

**METHODS:**

Qualitative research developed in 2019, in a primary health unit in the central region of the city of São Paulo, using participant observation and in-depth interviews with 16 health professionals and 8 older adults. The construction and interpretation of the narratives produced in the study were guided by the perspective of Gadamer’s philosophical hermeneutics and Ricoeur’s theory of interpretation. The theoretical framework of vulnerability/Careᵃ, as proposed by Ayres, guided the definition of the study and interpretative categories.

**RESULTS:**

Closely related to the difficulties, facilities and strategies to cope with the daily challenges in the health care of older adults, autonomy was an important marker of vulnerability (interpersonal, social and programmatic), indicating areas that require special attention, such as drug dispensing, urban mobility, social isolation, financial frailties and adequacy of service routines.

**CONCLUSION:**

Distinctly from an individual attribute, autonomy has proved to be the expression of relational characteristics, requiring plural and flexible practical-moral strategies, techniques and horizons, although always guided by the same ethical commitment to respect the singular needs of individuals.

## INTRODUCTION

Population aging is one of the biggest events in modern society. Due to the fall in fertility and mortality, and the consequent increase in life expectancy, virtually all regions of the globe, including Brazil, have experienced the gradual growth of the older adult population. It is estimated that, by 2050, about 1.5 billion people in the world will be 65 years of age or older, corresponding to approximately 16% of the population^1^^–^^3^.

From this perspective, since the 1980s, interest in older adults has grown, with worldwide encouragement to healthy or active aging, which has the autonomy of older adults among its premises^4^.

The first known publications that used the term “autonomy” date back to 1693. Derived from *alto* (self) and *nomia* (law or rule), the word was translated literally as “own rules.” However, despite the historical use, its meaning has no consensus in the context of health care^5^. It is currently recognized that autonomy involves dimensions that can be understood under the approaches of politics, sciences, powers or of individuals themselves, among others. Therefore, it is possible to speak of several autonomies^6^.

In a document from the Ministry of Health^6^ focused on the care of older adults, the meaning of autonomy is considered as “the individual’s ability to decide and command over their own actions, establishing and following their own convictions” (p. 80). Autonomy considered as the principle of bioethics, as shown by Beauchamp and Childress — that is, as part of a conception of self-government that considers the existence of an individual free of control, interference or any limitation that prevents them from making informed choices — has been criticized by some authors for being insufficient to address the practical issues of daily life^7^. According to Cohen and Gobbetti^8^, the limit of an individual’s freedom occurs in the context of relations between their inner and outer world, so no one is fully autonomous. Campos and Campos^9^, in addition to stating that autonomy is not absolute, add that the relation between autonomy and health is a dynamic process, which implies gradual losses or acquisitions, “as if they were coefficients relative to a standard of the subject themselves or to established social and historical standards” (p. 670)^9^. The exercise of autonomy is therefore close to an ethics because it is always in a situation and involves some value judgment. As a consequence, there is no autonomy *a priori*, for all, nor for any situation, which makes it even more complex to understand autonomy and its preservation^9^^,^^10^.

Despite the recognition of the value of autonomy for well-being and the encouragement in global policies to preserve the autonomy of the elderly, Lothian and Philip^11^, in a review study, state that although health services should have as priorities preserving dignity and autonomy and minimizing the suffering of patients, in many circumstances these goals are not achieved.

Considering the challenges mentioned, this study seeks to understand the relations between autonomy as a value and the challenges of health care for the elderly in the daily work of primary health care.

## METHODS

The aspects discussed here are part of a qualitative investigation developed in 2019 in a basic health unit (BHU) in the central region of the city of São Paulo, which used as tools participant observation in the service and in-depth interviews with professionals and patients^12^.

Individuals aged 60 years or older were considered older adults. To obtain greater heterogeneity and plurality of experiences, we sought to interview older adults with varying age, sex, functionality (independent and partially or totally dependent for daily living activities), income, family composition, race, religion, housing situation and relationship with the public service (exclusive use or concomitant with supplementary health). Older adults with these profiles were selected based on the indication of health teams or identification during participant observation. As for the professionals, the selection favored variation of age and team (capturing participants from all family health teams), time of service, sex and nationality. Considering the selection criteria, the invitation was made by the first author of this study directly to professionals. Once the invitation was accepted by professionals and older adults, an interview was scheduled at the place and time of the interviewee’s choice. Before the interview, the participants were oriented about the objectives of the study, reading and signing the informed consent form.

The final composition of the subjects of the study was determined by the saturation criterion^12^, that is, the 24 participants were defined by the researchers’ assessment achieving a diversity of respondents, which was capable of capturing the main profiles of interest to the research, and with sufficient empirical justification to respond consistently to the objectives of the study.

For participant observation, we used a script and a field diary in which observations, personal impressions and conversations in the field were recorded. Observing the interactions between older adults, family and professionals (among themselves and with patients) was privileged during individual visits, home visits, groups, team meetings and in the territory in which older adults and BHU were inserted.

Participant observation always carries the risk of interference arising from the presence of the researcher in the observed activities. Although it is impossible to definitively rule out this risk, we sought to avoid interpretative misconceptions arising from this situation. The progressive “dilution” of the observer’s interference by the constant and non-intrusive presence in the studied environment and, especially, the comparison of the observations with the content of the interviews and the analytical triangulation between the authors were relevant to obtain greater interpretative accuracy.

The comprehensive-interpretive process^13^, which supports the validity claim of this study, was based on the following steps: comprehensive reading of the interviews and the records of direct observation, objective-impregnation, insight, and understanding of the characteristics, identification, and analysis of the elements that are highlighted in the speeches, and the ideas in them, exploring the meanings that were seized at when the concerns of the researchers and the narratives that are produced met in relation to the autonomy of older adults and their implications for health care from the perspective of vulnerability.

The construction of the narratives of the interviews, the recording of field observations and the production of interpretative syntheses followed the movement of the so-called “hermeneutic arc.” The result of the study was produced by a progressive and dialectical movement between comprehensive totalizations, references that guided the interpretation of the findings and thorough analysis of the empirical material, which, with the help of the literature already produced, enriched understanding, configuring the “virtuous circularity” between whole and part, proper to the hermeneutic perspective^14^.

The empirical material was constructed from two thematic axes guided by the theoretical framework of vulnerability/care: 1) recognizing the meanings of autonomy for the deponents; and 2) understanding the main difficulties found in care in relation to the autonomy of older adults.

The concept of vulnerability, as developed by Ayres^15^ based on the original proposition of Mann and Tarantola^16^, establishes a set of individual and collective characteristics related to the greater susceptibility of individuals and communities to an event and, in association, less availability of resources for their protection. Ayres shows three interconnected dimensions within the framework of vulnerability: individual, social and programmatic. The individual dimension concerns the individual’s own factors and their interpersonal interactions, ranging from the health condition to values and way of life of that person. The social concerns inseparable contextual aspects of the previous dimension, such as gender relations, race relations, access to health, education, justice and profession, among others. Finally, the programmatic dimension of vulnerability analyses highlights how institutions act as elements that reduce, reproduce or increase the vulnerability of individuals in given social circumstances.

As a correspondent, in the action plan, thus integrating the apprehension of health conditions through vulnerability, the concept of health Care points to the need to articulate the dimension of *technical efficacy* (instrumental sense of actions based on health technosciences) with that of *practical success* (sense of the purposes and means of actions in front of the values and interests of patients in the concrete contexts of their daily lives). The effectiveness of such articulation depends on considering as a normative reference of health actions, in addition to morphofunctional normality, the so-called “happiness project” of people, what moves them and gives meaning to their existences^17^.

The study was approved by the Research Ethics Committee of the institutions involved, and the certificates of presentation for ethical appreciation (CAAE) in the Plataforma Brasil are, respectively, 90497018.1.0000.0065 and 90497018.1.3001.5479.

## RESULTS AND DISCUSSION

The health unit studied had 13,786 registered patients, of whom 9,708 were linked to three family health teams. Approximately 19.7% of the total population in the service was over 60 years old.

We interviewed 8 older adults (whose main characteristics are summarized in Chart 1) and 16 health professionals aged between 29 and 67 years, who had been working in the service from 1 month to 17 years, three of them male and the rest female. One interviewee declared herself black, four brown and the others white. Fourteen of the professionals interviewed comprised the family health teams: 7 community health agents, 2 nursing technicians, 3 nurses, 2 family and community physicians (FCP). Two others — a geriatrician and a social worker — were integrated into BHU as required by management.

**Chart:** Main characteristics of the older adults interviewed.

Older adult	Sex	Age (years)	Marital status	Education	Religion	Work situation	Household nucleus	Housing situation	Income (R$)
Iara	F	84	Widow	Incomplete elementary school	Catholic	Pensioner	Son (older adult)	Own apartment	R$ 4,654.20
Sônia	F	74	Single	Higher education	Catholic	Retired (disability + age), active craftsman	Sister (older adult)	Own house	R$ 4,654.20
Carmen	F	90	Single	Higher education	Catholic	Retiree	Alone	Own apartment	Not informed
João	M	81	Married	Higher education	Atheist	Retiree	Wife (older adult)	Own apartment	R$ 2,400.00
Dirce	F	82	Widow	Illiterate	Protestant	Continuous Cash Benefit	Brother (older adult)	Own house	R$ 700.00
Ivete	F	75	Divorced	Elementary school	Catholic	Retired, active seamstress	Daughters (two)	Rented house	R$ 2,499.00
Rute	F	90	Widow	Higher education	Catholic	Retiree	Alone	Own house	R$ 3,500.00
Joaquim	M	89	Divorced	Illiterate	Protestant	Retired due to disability	Alone	CDHU housing	R$ 1,600.00

F: Female, M: Male. CDHU: *Companhia de Desenvolvimento Habitacional e Urbano.*

### What autonomy are we talking about?

Even if we consider autonomy essential for care — as well as a value reinforced by legislation, national and international policies — its definition has no consensus, particularly in the field of health^5^^–^^8^. Among the health professionals interviewed, autonomy was sometimes valued in the field of functionality, such as independence for carrying out activities of daily living, sometimes as decision-making capacity.

*Going to the market, paying the bills, taking a shower alone. There are some who can’t even do that, right? Unfortunately. Preparing food by yourself, going to the movies. Doing whatever you want, regardless of people. Alone or accompanied, but without depending on someone.* (Carolina, nurse)

*Unlike autonomy, which is more a matter of decision-making ability, even if you are physically dependent on another individual, you have your autonomy, your full ability to decide what you want to be done for your life or not.* (Daniela, geriatrician)

Understanding autonomy as a decision-making capacity (or judgment) is one of the main criteria considered by health professionals (and by judges, in judicialized cases) for decision-making in the health follow-up of older adult patients. Several situations involving different perceptions of autonomy generate anxiety in the team, such as when the older adult refuses some intervention that health professionals believe is the best (when considering independence or functional capacity).

*These cases, when the older adult wants absolutely nothing, are the cases that most cause anxiety and stress in people, in the team. Because we become a spectator of a show that’s not very nice. When we try to discuss the cases with the prosecutor, he says the following: to what extent do you consider him incapable? None. So he will decide whatever he wants to be done*. (Deborah, social worker)

The possibility of performing tasks independently is also expressed by some older adults as autonomy and as a value for old age. Needing help for some activities is one of the main limitations faced in everyday life.

*I already have trouble getting out, walking alone, my son needs to stop working to take me to places. I feel sorry that I’m not able to do it, that I have to ask a person who works all day and then comes home and needs to do other things that I could do.* (Iara, 84 years old)

On the other hand, if physical dependence generates some possibility of exchange, if the elderly understand that they can help other people, even because of their dependence, this is accepted more easily.

*If I’m going to ask a street sweeper for [help], it seems to me is that they are thanking me for asking, they’re so delicate. They drop everything, drop the broom and take me by the hand, it’s fantastic, it’s a delight.* (Sônia, 74 years old)

### The Autonomy of Older Adults in the Daily Life of Primary Health Care

When analyzed in the concrete context of relationships, the question of autonomy shows its complexity. It cannot be delimited only as functionality or decision-making capacity, because, at the interface between power and decision-making, factors inherent in personal history, culture, social environment, technology and access to programs and policies will give different meanings to these terms, with different implications on health-disease-care processes. Due to its relation to different horizons of life, in team meetings, appointments, groups and reception, the issues pertinent to autonomy and its preservation in older adults arise in several “problem situations” that acquire different colors in each context. Among the various difficulties encountered in the care of older adults in primary care, the dilemmas related to autonomy refer to vulnerabilities that extrapolate the strictly individual problem of the functional capacity of an older adult. The question becomes: incapacity or limitation, of action or decision, facing what challenges?

Non-adherence to prescriptions is considered one of the main problems in the care of the elderly in the reports of the deponents. However, factors such as illiteracy, low socioeconomic status, beliefs, ignorance or even forgetfulness are shown as involved in the difficulty of adherence, highlighting the perception that non-adherence is due to several factors, in addition to the older adult’s own will.

*Many of these older adults can’t keep up with what we are talking about, they can’t actually see what is true for them there. There are some who mix religion “no, I won’t take it because God will heal me.”* (…) *Many say that they receive minimum wage, that sometimes they have to buy medicine, we often don’t have that medicine here.* (Diego, nursing technician)

This perception is compatible with the current literature, which adds, in addition to these factors, that the amount of medications, weaknesses in the drug supply network, substance abuse and the presence or absence of family support also influence adherence^6^^,^^18^^,^^19^.

In the field of morphofunctionality, the most common difficulties were gait or locomotion, but urinary incontinence, decreased visual acuity and cognitive deficit or forgetfulness were also mentioned. However, once again, along with physical limitation, situations in the city context (such as violence and poor adaptation of urban spaces) appear in the reports that qualify these complaints and also set limits for the performance of care, interfering, for example, in the practice of physical activities or in the search for the BHU. Limitation of older adults or of public space? Disability of the elderly or difficulties with an unprepared environment for diversity?

*Locomotion or even… It’s not always locomotion. Sometimes the person has no security to leave the house. My area is very close [to the unit]. But I have a patient who doesn’t come here because he’s afraid to cross the avenue. Because he doesn’t feel safe. Because he doesn’t feel protected. Because, in a street with a traffic light that is green for 30 seconds, the person is afraid of not having time to cross. Yes, they’re things so simple, but they very much limit the older adult.* (Andrea, FCP)

According to a study based on the ELSI-Brasil database^20^, 23.2% of individuals over 50 years of age have limitations for basic activities of daily living, especially in relation to mobility at home and to dress. Locomotion problems are common in the elderly and can result not only from alterations in the lower limbs, but also from cognitive deficits, alterations in sensoperception and environmental factors^21^^,^^22^. Ferreira et al.^23^ also demonstrate that urban mobility is essential to facilitate or undermine social participation, in particular of older people, who are particularly sensitive to these characteristics of their surroundings and may favor social exclusion.

Among the challenges mentioned by the teams, several narratives referred to cases of social isolation of older adults, both due to their own desire (even when assessed as necessary care by other people) and by family incontinence or insufficiency.

*Friends walk away, that’s hard too. We say, “there were so many people,” you move to a different place and it seems that you went abroad, not to the countryside, abroad. You don’t get a phone call, you don’t get a visit, a letter, nothing.* (Iara, 84 years old)

*This is typical of age, I know, what I need is people, people inside the house.* (Rute, 90 years old)

As in the interviews, literature points out that social isolation can intervene in various ways in well-being. Social ties provide essential support in times of illness, encourage people to adopt better health habits and impact the functioning of the immune system. In addition, the absence of these ties may imply cognitive impairment and poor self-perception of health, increased mortality and reduced practice of healthy activities or ability to survive a natural disaster^24^^,^^25^. Family availability is also considered a protective factor for aging, since loneliness, depression and poor health conditions are related to the absence of kinders^24^.

The team and the older adults also described situations in which financial condition was decisive for care relationships. In some cases, the older adult was the main provider, so the needs of the family guided the allocation of their resources. In others, the family’s low-income condition limited the older adult’s access to the service, due to lack of transportation resources or even for fear of walking in an environment marked by violence, either to go to the service or to exercise.

*They did want to go out, but the fear of leaving… Some people say, “oh, I can’t go two, three blocks away anymore, I’m afraid because there’s so much robbery here on the street” and you know, stuff like that. And there are no places of leisure for them, it’s far from here.* (Olivia, community health agent)

Barbosa, Oliveira and Fernandes^26^ show that, in fact, a poor and deprived neighborhood is associated with worse cardiovascular health, increased incidence of infectious diseases, prevalence of depression and functional limitation, worse self-assessment of health and interference in access to health equipment and services.

Difficulties in the care of the elderly in primary care also refer to the programmatic dimension of vulnerability of older adults, related to access and accessibility (geographical issues, excess demand, short time for care, distant appointments), excess bureaucracy, lack of computerization, ignorance of the entire network of public services aimed at older adults or even incompatibility between supply and demand for these network services (such as day centers, reference units for the health of older adults, etc.).

*It’s unbelievable that in 2019 we don’t have an electronic medical record yet. This is shocking, you spend more time on papers and papers and we don’t have reliable data on incidence, prevalence of disease, what we are improving in the population, whether we are really improving the health of our population, care, in general. It is essential to computerize, so that we’ll be able to make a better network connection. I think the devices are all there, but they are poorly connected, in general. And this lack of communication from the services makes it difficult. For example, the patient arrives in the HC* [Hospital das Clínicas]*, tertiary service, they have no connection to the network, you can’t return the HC patient to primary care, for example, and there the patient is being treated without indication in the tertiary service.* (Daniela, geriatrician)

This type of limitation is not exclusive to this BHU, and part of what is attributed as low autonomy of older adults, or an exercise of autonomy that limits the action of services, needs to be reinterpreted in light of what is actually being offered to older adults as a possibility of care. In fact, excessive demand, large population linked to the teams, delay in scheduling, bureaucracy, lack of computerization and few resources are problems pointed out in general by patients and health professionals^3^^,^^27^^,^^28^^,^^29^.

## FINAL REMARKS

The generalization of findings of a qualitative research should be examined very carefully, because the singularization of the examined situation, necessary for interpretative deepening, competes with the extensiveness of the categories with which the study is constructed. What is expected is that the hermeneutic fecundity of the comprehensible framework produced can be useful to other comparable contexts.

The results shown here will be more easily transferable to the context of older adults living in large urban centers, in industrialized Western societies, in social segments of middle and lower-middle income, with access to primary health care services and some degree of social support via public policies. This does not mean, however, that the study is irrelevant to other contexts. The way in which the relations between autonomy and health-disease-care processes of older adults have been configured here may help to reconfigure the understanding of health care challenges for these people in different contexts. In this sense, criticism of individualizing readings of the value of autonomy and, on the contrary, emphasis on its analytical power on related social and programmatic practices may be particularly useful, not only for older adults, but also for other situations in which the issue of autonomy is relevant to care, such as in the case of people with disabilities or mental disorders.

According to the above, in the context of primary care, the difficulties found in the care of older adults permeate the autonomy embodied with the vulnerabilities to which each elderly person is exposed at a given time. Looking at the care needs of older adults through a relational and procedural perspective of their limits is a potential way to see autonomy not as an end in itself, much less as an obstacle or resistance that needs to be overcome to protect older adults from the risks brought by aging, but rather as a marker of *what* and *how* it needs to be transformed (individually, socially and programmatically) for an effective care, that is, to mobilize technical resources that enhance practical successes in terms of health.

The greater or lesser autonomy that one or another individual claims, in this or that aspect of their daily lives, actually signals regions of vulnerability, relational areas (interpersonal, social, programmatic) that require special attention so that technical efficacy and practical success may indeed interact constructively and adequately to the happiness projects of older adults.

By being constructed and understood only in the concrete contexts of the daily interaction of older adults, autonomy is gaining different features as a result of the different situations of practice, which requires thinking of it not as an individual attribute (which the older adult *still has* or *has already lost*), but as a marker of relational characteristics that demand technical strategies and plural and flexible practical-moral horizons, always from an ethical horizon of respect for the other and responsibility in relation to it.
