# Supplementary material for: Older adult health and primary care: autonomy, vulnerabilities and challenges of care
Source: Rev Saude Publica. 2021 May 6;55:29. doi: 10.11606/s1518-8787.2021055002856 (PMC8102029; doi:10.11606/s1518-8787.2021055002856)
Supplement: Translation

# Translation: Saúde do idoso e atenção primária: autonomia, vulnerabilidades e os desafios do cuidado

**Authors:** Jeane Lima e Silva Carneiro, José Ricardo de Carvalho Mesquita Ayres

**Affiliations:** I São PauloSP Brasil Universidade São Paulo. Faculdade de Medicina da Universidade de São Paulo. Programa de Pós-Graduação em Saúde Coletiva, São Paulo, SP, Brasil; II São PauloSP Brasil Universidade de São Paulo. Faculdade de Medicina da Universidade de São Paulo. Departamento de Medicina Preventiva, São Paulo, SP, Brasil

**Keywords:** Idoso, Autonomia Pessoal, Vulnerabilidade em Saúde, Processo Saúde-Doença, Envelhecimento, Atenção Primária à Saúde

## Abstract

**OBJETIVO:**

Compreender as relações entre a autonomia e processos de saúde-doença-cuidado do idoso no cotidiano da atenção primária à saúde.

**MÉTODOS:**

Investigação qualitativa desenvolvida em 2019, em uma unidade básica de saúde da região central da cidade de São Paulo, utilizando observação participante e entrevistas em profundidade com 16 profissionais de saúde e 8 idosos. A construção e interpretação das narrativas produzidas no estudo foram orientadas pela perspectiva da hermenêutica filosófica de Gadamer e a teoria da interpretação de Ricoeur. O quadro teórico da vulnerabilidade/Cuidadoᵃ, tal como proposto por Ayres, orientou a definição do desenho do estudo e das categorias interpretativas.

**RESULTADOS:**

Estreitamente relacionada às dificuldades, facilidades e estratégias de enfrentamento dos desafios diários na atenção à saúde do idoso, a autonomia mostrou-se um importante marcador de vulnerabilidade (interpessoal, social e programática), indicando áreas que requerem atenção especial, como dispensação de medicamentos, mobilidade urbana, isolamento social, fragilidades financeiras e adequação das rotinas dos serviços.

**CONCLUSÃO:**

Distintamente de um atributo individual, a autonomia revelou-se expressão de características relacionais, exigindo estratégias, técnicas e horizontes prático-morais plurais e flexíveis, embora sempre orientados pelo mesmo compromisso ético de respeito às necessidades singulares dos indivíduos.

## INTRODUÇÃO

O envelhecimento populacional é um dos maiores acontecimentos da sociedade moderna. Em função da queda da fecundidade e da mortalidade, e do consequente aumento da expectativa de vida, praticamente todas as regiões do globo, incluindo o Brasil, têm experimentado o crescimento gradual da população de idosos. Estima-se que em 2050 cerca de 1,5 bilhão de pessoas no mundo terão 65 anos ou mais, correspondendo a aproximadamente 16% da população^1^^–^^3^.

A partir deste panorama, desde os anos 1980 há um crescente interesse no idoso, com o estímulo em todo o mundo ao envelhecimento saudável ou ativo, que tem a autonomia do idoso entre suas premissas^4^.

As primeiras publicações conhecidas que usaram o termo “autonomia” datam de 1693. Derivada de *auto* (próprio) e *nomia* (norma ou regra), a palavra foi traduzida literalmente, como “regras próprias”. Contudo, apesar do uso histórico, não há consenso quanto a seu significado no contexto do cuidado em saúde^5^. Atualmente se reconhece que a autonomia envolve dimensões que podem ser compreendidas sob os enfoques da política, das ciências, dos poderes ou do próprio indivíduo, dentre outros. Por isso, é possível falar em diversas autonomias^6^.

Em documento do Ministério da Saúde^6^ voltado ao cuidado do idoso, o significado de autonomia é tomado como “a capacidade do indivíduo de decisão e comando sobre as próprias ações, estabelecendo e seguindo as próprias convicções” (p. 80). A autonomia considerada como princípio da bioética, tal como apresentada por Beauchamp e Childress – isto é, como parte de uma concepção de autogoverno que considera a existência de um indivíduo livre de controle, interferência ou qualquer limitação que o impeça de fazer escolhas esclarecidas – tem sido criticada por alguns autores por ser insuficiente para abordar as questões práticas do cotidiano^7^. Segundo Cohen e Gobbetti^8^, o limite da liberdade de um indivíduo se dá no contexto de relações entre seu mundo interno e o externo, assim ninguém é totalmente autônomo. Campos e Campos^9^ além de afirmarem que a autonomia não é absoluta, acrescentam que a relação entre autonomia e saúde é um processo dinâmico, que implica perdas ou aquisições gradativas, “como se fossem coeficientes relativos a um padrão do próprio sujeito ou a padrões sociais e históricos estabelecidos”(p. 670)^9^. O exercício da autonomia se aproxima, portanto, de uma ética, pois sempre se coloca em situação e envolve algum juízo de valor. Como consequência, não há uma autonomia *a priori*, para todos e todas, nem para qualquer situação, o que torna ainda mais complexo entender a autonomia e sua preservação^9^^,^^10^.

Apesar do reconhecimento do valor da autonomia para o bem-estar e do incentivo em políticas mundiais para preservar a autonomia dos idosos, Lothian e Philip^11^, em um estudo de revisão, afirmam que, embora os serviços de saúde devam ter como prioridades preservar a dignidade e a autonomia e minimizar o sofrimento dos pacientes, em muitas circunstâncias esses objetivos não são atingidos.

Considerando os desafios apontados, o presente estudo busca compreender as relações entre a autonomia como valor e os desafios do cuidado à saúde do idoso no cotidiano de trabalho da atenção primária à saúde.

## MÉTODOS

Os aspectos aqui discutidos são parte de uma investigação qualitativa desenvolvida em 2019 em uma Unidade Básica de Saúde (UBS) da região central da cidade de São Paulo, que utilizou como ferramentas a observação participante no serviço e entrevistas em profundidade com profissionais e usuários^12^.

Foram considerados idosos os indivíduos com 60 anos ou mais. Para obter maior heterogeneidade e pluralidade de vivências, buscou-se entrevistar idosos com variação de idade, sexo, funcionalidade (independentes e parcial ou totalmente dependentes para atividades de vida diária), renda, composição familiar, raça, religião, situação de moradia e relação com o serviço público (uso exclusivo deste ou concomitante com a saúde suplementar). Idosos com esses perfis foram selecionados a partir de indicação das equipes de saúde ou de identificação durante a observação participante. Quanto aos profissionais, a seleção privilegiou variação de idade e de equipe (captando depoentes de todas as equipes de saúde da família), tempo de atuação no serviço, sexo e nacionalidade. Considerando os critérios de seleção, o convite foi realizado pela primeira autora deste estudo diretamente aos profissionais. Uma vez aceito o convite por profissionais e idosos, foi agendada entrevista em local e horário de escolha do entrevistado. Antes da entrevista, os depoentes foram orientados sobre os objetivos do estudo, lendo e assinando o termo de consentimento livre e esclarecido.

A composição final dos sujeitos do estudo foi determinada por critério de saturação^12^, isto é, os 24 participantes foram definidos pela avaliação dos pesquisadores de que se alcançara uma diversidade de entrevistados capaz de captar os principais perfis de interesse para a pesquisa, e de que havia suficiente fundamentação empírica para responder de forma consistente aos objetivos do estudo.

Para a observação participante, utilizou-se um roteiro e um diário de campo em que foram registradas observações, impressões pessoais e conversas no campo. Foi privilegiada a observação das interações entre idosos, familiares e profissionais (entre si e com os usuários) durante atendimentos individuais, visitas domiciliares, grupos, reuniões de equipe e no território em que idosos e UBS estavam inseridos.

A observação participante traz sempre o risco de interferências decorrentes da presença do pesquisador nas atividades observadas. Embora seja impossível afastar em definitivo esse risco, buscou-se evitar vieses interpretativos decorrentes dessa situação. A observação participante sempre produz interferências decorrentes da presença do pesquisador nas atividades observadas. Embora seja impossível evitá-las, é importante evitar equívocos interpretativos decorrentes da situação. A progressiva “diluição” da interferência do observador pela presença constante e não intrusiva no ambiente estudado e, especialmente, o cotejamento das observações com o conteúdo das entrevistas e a triangulação analítica entre os autores mostraram-se relevantes para obter-se maior acurácia interpretativa.

O processo compreensivo-interpretativo^13^, que sustenta a construção e a busca de validade deste estudo se deu nas seguintes etapas: leitura compreensiva dos depoimentos e dos registros de observação participante, objetivando impregnação, visão de conjunto e apreensão das particularidades; identificação e problematização dos elementos destacados nos discursos e das ideias presentes; exploração dos significados apreendidos no encontro entre as preocupações dos pesquisadores e as narrativas produzidas no que se refere à autonomia do idoso e suas implicações para a atenção à saúde na perspectiva da vulnerabilidade.

A construção das narrativas das entrevistas, o registro das observações de campo e a produção de sínteses interpretativas seguiu o movimento do chamado “arco hermenêutico”. O resultado do estudo foi sendo produzido por um movimento progressivo e dialético entre totalizações compreensivas, referências que norteavam a interpretação dos achados e análise minuciosa do material empírico, que, com a ajuda da literatura já produzida, ia enriquecendo a compreensão, configurando a “circularidade virtuosa” entre todo e parte, própria da perspectiva hermenêutica^14^.

O material empírico foi construído a partir de dois eixos temáticos orientados pelo quadro teórico da vulnerabilidade/cuidado: 1) reconhecimento dos significados de autonomia para os depoentes; e 2) compreensão das principais dificuldades encontradas no cuidado em relação à autonomia do idoso.

O conceito de vulnerabilidade, tal como desenvolvido por Ayres^15^ com base na proposição original de Mann e Tarantola^16^, estabelece um conjunto de características individuais e coletivas relacionadas à maior suscetibilidade de indivíduos e comunidades a um evento e, associadamente, menor disponibilidade de recursos para sua proteção. Ayres apresenta três dimensões interligadas dentro do quadro da vulnerabilidade: individual, social e programática. A individual concerne a fatores próprios do indivíduo e suas interações interpessoais, que vão desde a condição de saúde até valores e modo de vida dessa pessoa. A social diz respeito a aspectos contextuais indissociáveis da dimensão anterior, como relações de gênero, relações raciais, acesso à saúde, educação, justiça e profissão, dentre outros. Por fim, a dimensão programática das análises de vulnerabilidade destaca o modo como as instituições atuam como elementos que reduzem, reproduzem ou aumentam a vulnerabilidade dos indivíduos em dadas circunstâncias sociais.

**Quadro 1:** Principais características dos idosos entrevistados.

Idoso	Sexo	Idade (anos)	Estado marital	Escolaridade	Religião	Situação trabalhista	Núcleo domiciliar	Situação de moradia	Renda (R$)
Iara	F	84	Viúva	Fundamental incompleto	Católica	Pensionista	Filho (idoso)	Apartamento próprio	R$ 4.654,20
Sônia	F	74	Solteira	Superior completo	Católica	Aposentada (invalidez + idade), artesã ativa	Irmã (idosa)	Casa própria	R$ 4.654,20
Carmen	F	90	Solteira	Superior completo	Católica	Aposentada	Sozinha	Apartamento próprio	Não informado
João	M	81	Casado	Superior completo	Ateu	Aposentado	Esposa (idosa)	Apartamento próprio	R$ 2.400,00
Dirce	F	82	Viúva	Não alfabetizada	Protestante	Benefício de Prestação Continuada	Irmão (idoso)	Casa própria	R$ 700,00
Ivete	F	75	Divorciada	Fundamental completo	Católica	Aposentada, costureira ativa	Filhas (duas)	Casa alugada	R$ 2.499,00
Rute	F	90	Viúva	Superior completo	Católica	Aposentada	Sozinha	Casa própria	R$ 3.500,00
Joaquim	M	89	Divorciado	Não alfabetizado	Protestante	Aposentado por invalidez	Sozinho	Moradia CDHU	R$ 1.600,00

F: Feminino, M: Masculino. CDHU: Companhia de Desenvolvimento Habitacional e Urbano.

Como correspondente, no plano da ação, deste modo integrador da apreensão das condições de saúde por meio da vulnerabilidade, o conceito de Cuidado em saúde aponta para a necessidade de articular a dimensão de *êxito técnico* (sentido instrumental das ações baseadas nas tecnociências da saúde) com a de *sucesso prático* (sentido das finalidades e meios das ações frente aos valores e interesses dos pacientes nos contextos concretos de seu cotidiano). Para a efetividade de tal articulação, aponta-se a necessidade de tomar como referência normativa das ações de saúde, para além da normalidade morfofuncional, o chamado “projeto de felicidade” das pessoas, aquilo que as move e dá sentido a suas existências^17^.

O estudo foi aprovado pelo Comitê de Ética em Pesquisa das instituições envolvidas, e os Certificados de Apresentação para Apreciação Ética (CAAE) na Plataforma Brasil são, respectivamente, 90497018.1.0000.0065 e 90497018.1.3001.5479.

## RESULTADOS E DISCUSSÃO

A unidade de saúde estudada possuía 13.786 usuários adscritos, dos quais 9.708 estavam vinculados a três equipes de saúde da família. Aproximadamente 19,7% da população total atendida no serviço tinha mais de 60 anos.

Foram entrevistados 8 idosos (cujas principais características estão sintetizadas no Quadro) e 16 profissionais de saúde com idade entre 29 e 67 anos e tempo de atuação no serviço de 1 mês a 17 anos, três deles do sexo masculino e os demais do feminino. Uma entrevistada se declarou negra, quatro pardos e os demais brancos. Catorze dos profissionais entrevistados compunham as equipes de saúde da família: 7 agentes comunitários de saúde, 2 técnicos de enfermagem, 3 enfermeiros, 2 médicos de família e comunidade (MFC). Outros dois – uma geriatra e um assistente social – eram integrados à UBS conforme demanda da gestão.

### De qual Autonomia se Fala?

Mesmo considerando que a autonomia é essencial para o cuidado – além de valor reforçado pela legislação, políticas nacionais e internacionais –, não há um consenso sobre sua definição, particularmente no campo da saúde^5^^–^^8^. Dentre os profissionais de saúde entrevistados, a autonomia ora era valorizada no campo da funcionalidade, como a independência para realização de atividades de vida diária, ora como capacidade de decisão.

*Ir ao mercado, pagar as contas, tomar banho sozinho. Tem uns que não conseguem nem fazer isso, né? Infelizmente. Preparar comida sozinho, ir ao cinema. Fazer tudo o que quer, independente das pessoas. Sozinho ou acompanhado, mas sem depender de alguém.* (Carolina, enfermeira)

*Diferente de autonomia, que é uma questão muito mais de capacidade de decisão, mesmo que você dependa fisicamente de outro indivíduo, você tem sua autonomia, sua capacidade plena de decisão do que você quer que seja feito para sua vida ou não.* (Daniela, geriatra)

O entendimento da autonomia como a capacidade de decisão (ou julgamento) é um dos principais critérios considerados pelos profissionais de saúde (e pelos juízes, nos casos judicializados) para a tomada de decisões no seguimento de saúde de pacientes idosos. Diversas situações envolvendo percepções diversas de autonomia geram angústia na equipe, como quando o idoso recusa alguma intervenção que os profissionais de saúde entendem ser a melhor (ao se considerar a independência ou capacidade funcional).

*Esses casos, quando o idoso não quer absolutamente nada, são os casos que mais causam ansiedade e estresse na gente, na equipe. Porque a gente vira espectador de um espetáculo que não é muito legal. Quando a gente tenta discutir os casos com o Ministério Público, ele diz o seguinte: em que medida você considera ele incapaz? Nenhuma. Então tudo que ele decidir ele vai decidir*. (Débora, assistente social)

A possibilidade de realizar tarefas de forma independente também é expressa por alguns idosos como autonomia e como um valor para a velhice. Precisar de ajuda para algumas atividades é uma das principais limitações enfrentadas no cotidiano.

*Já tenho problema de sair, de andar sozinha, meu filho precisa largar de trabalhar para me levar. Eu fico sentida só, assim, de não poder fazer, de precisar pedir para uma pessoa que trabalha o dia inteiro e depois chega em casa e precisa fazer outras coisas que eu poderia fazer.* (Iara, 84 anos)

Por outro lado, se a dependência física gera alguma possibilidade de troca, se o idoso entende que pode ajudar a outras pessoas, mesmo em razão da sua dependência, esta é aceita com maior facilidade.

*Se eu vou pedir para um varredor de rua, a impressão que me dá é que eles estão agradecendo de eu pedir, de tão delicado. Eles largam tudo, largam a vassoura e me pegam pela mão, é fantástico isso, é uma delícia.* (Sônia, 74 anos)

### A Autonomia do Idoso no Cotidiano da Atenção Primária à Saúde

Quando analisada no contexto concreto das relações, a questão da autonomia mostra sua complexidade. Ela não admite ser delimitada apenas como funcionalidade ou capacidade de decisão, porque, na interface entre o poder e o decidir fazer, fatores inerentes à história pessoal, cultura, meio social, tecnologia e acesso a programas e políticas vão conferir sentidos diversos a estes termos, com diferentes implicações sobre os processos de saúde-doença-cuidado. Por sua relação com diversos horizontes da vida, em reuniões de equipe, atendimentos, grupos e acolhimento, as questões pertinentes à autonomia e sua preservação no idoso surgem em diversas “situações-problemas” que adquirem cores diferentes em cada contexto. Dentre as diversas dificuldades encontradas no cuidado do idoso na atenção primária, os dilemas relacionados à autonomia remetem a vulnerabilidades que extrapolam a problemática estritamente individual da capacidade funcional do idoso. A questão passa a ser: incapacidade ou limitação, de ação ou decisão, frente a que desafios?

A não adesão às prescrições é considerada um dos principais problemas no cuidado dos idosos nos relatos dos depoentes. Porém, fatores como analfabetismo, baixa condição socioeconômica, crenças, desconhecimento ou mesmo esquecimento são apresentados como envolvidos na dificuldade de adesão, ressaltando-se a percepção de que a não adesão se deve a diversos fatores, para além da própria vontade do idoso.

*Muitos desses idosos não conseguem acompanhar o que a gente fala, não conseguem ver de fato o que é verdadeiro para eles ali. Tem uns que misturam religião “não, não vou tomar não porque Deus me cura”.* (…) *Muitos falam que recebem salário-mínimo, que às vezes têm que comprar remédio, muitas vezes o remédio não tem aqui.* (Diego, técnico de enfermagem)

Tal percepção é compatível com a literatura corrente, que acrescenta, além desses fatores, que a quantidade de medicações, as debilidades na rede de fornecimento dos medicamentos, abuso de substâncias e presença ou não de apoio familiar também influenciam na adesão^6^^,^^18^^,^^19^.

No campo da morfofuncionalidade, as dificuldades mais encontradas foram as de marcha ou de locomoção, mas também foram citadas incontinência urinária, diminuição da acuidade visual e déficit cognitivo ou esquecimento. Porém, mais uma vez, junto à limitação física, aparecem nos relatos situações do contexto da cidade (como violência e pouca adaptação dos espaços urbanos) que qualificam essas queixas e configuram também limites para a realização do cuidado, interferindo, por exemplo, na prática de atividades físicas ou na procura pela UBS. Limitação do idoso ou do espaço público? Incapacidade do idoso ou dificuldades com um meio despreparado para a diversidade?

*Locomoção ou até… Nem sempre é locomoção. Às vezes, a pessoa não tem segurança de sair de casa. A minha área é muito perto. Mas tenho paciente que não vem até aqui, porque tem medo de atravessar a avenida. Porque não se sente seguro. Porque não se sente protegido. Porque, pra atravessar a rua do farol que fica aberto 30 segundos, a pessoa tem medo de não dar tempo. Sim, são coisas tão simples, mas que limitam muito a pessoa idosa.* (Andrea, MFC)

Segundo estudo derivado da base de dados do ELSI-Brasil^20^, 23,2% dos indivíduos com mais de 50 anos apresentam limitações para atividades básicas de vida diária, sobretudo em relação à mobilidade em casa e para se vestir. Os problemas de locomoção são frequentes em idosos e podem decorrer não só de alterações próprias dos membros inferiores, mas também de déficit cognitivo, alterações de sensopercepção e fatores ambientais^21^^,^^22^. Ferreira et al.^23^ também demonstram que a mobilidade urbana é essencial para facilitar ou prejudicar a participação social em especial dos mais velhos, que são particularmente sensíveis a estas características do seu entorno, e podem favorecer a exclusão social.

Dentre os desafios referidos pelas equipes, várias narrativas remetiam a casos em que havia isolamento social do idoso, tanto por desejo próprio (mesmo quando avaliado como necessário o cuidado por outras pessoas) quanto por incontinência ou insuficiência familiar.

*Os amigos se afastam, isso é duro também. A gente fala “poxa, tinha tanta gente”, você muda de local parece que você foi embora para o exterior, não interior, exterior. Você não recebe um telefonema, você não recebe uma visita, uma carta, nada.* (Iara, 84 anos)

*Isso é próprio da idade, eu sei, o que faz falta pra mim é gente, gente dentro de casa.* (Rute, 90 anos)

Assim como nas entrevistas, a literatura assinala que o isolamento social pode intervir de diversas maneiras no bem-estar. Laços sociais fornecem suporte essencial em momentos de doença, incentivam as pessoas a adotar melhores hábitos de saúde e impactam no funcionamento do sistema imunológico. Além disso, a ausência desses laços pode implicar comprometimento cognitivo e autopercepção ruim da saúde, aumento de mortalidade e redução da prática de atividades saudáveis ou capacidade de sobreviver a um desastre natural^24^^,^^25^. A disponibilidade da família também é considerada um fator de proteção para o envelhecimento, uma vez que solidão, depressão e condições ruins de saúde estão relacionadas à ausência de parentescos^24^.

A equipe e os idosos também apresentaram situações em que a condição financeira foi determinante para as relações de cuidado. Em alguns casos, o idoso era o principal provedor, de forma que as necessidades da família orientaram a destinação dos seus recursos. Em outros, a condição de baixa renda da família limitou o acesso do idoso ao serviço, por falta de recursos para transporte ou até por receio de caminhar em um entorno marcado por violência, seja para ir ao serviço, seja para se exercitar.

*Eles até queriam sair, mas o medo de saírem… Alguns falam “ah, eu não consigo mais sair dois, três quarteirões, eu tenho medo porque tem tanto assalto aqui na rua” e sabe, todas essas coisas assim. E não tem um lugar de lazer para eles, é longe daqui.* (Olívia, agente comunitária de saúde)

Barbosa, Oliveira e Fernandes^26^ expõem que, de fato, uma vizinhança pobre e com privações está associada a pior saúde cardiovascular, aumento da incidência de doenças infecciosas, da prevalência de depressão e de limitação funcional, pior autoavaliação de saúde e interferências no acesso a equipamentos e serviços de saúde.

Dificuldades no cuidado do idoso na atenção primária também remetem à dimensão programática de vulnerabilidade de idosos, relativas a acesso e acessibilidade (questões geográficas, excesso de demanda, tempo curto para atendimento, agendamentos distantes), excesso de burocracia, falta de informatização, desconhecimento de toda a rede de serviços públicos voltados aos idosos ou mesmo incompatibilidade entre oferta e demanda por esses serviços da rede (como centros-dia, unidade de referência à saúde do idoso, etc).

*Não é possível em 2019 ainda não ter prontuário eletrônico. É chocante isso, você gasta maior tempo em papéis e papéis e a gente não tem dados fidedignos de incidência, de prevalência de doença, o que que a gente está melhorando na população, se a gente está realmente melhorando a saúde da nossa população, de assistência, de forma geral. É essencial informatizar, que a gente vai conseguir fazer uma conexão melhor da rede. Eu acho que os dispositivos todos estão aí, mas eles são mal conectados, de forma geral. E essa falta de comunicação dos serviços que dificulta. Por exemplo, chega o paciente lá no HC* [Hospital de Clínicas]*, serviço terciário, eles não têm conexão nenhuma com a rede, você não consegue devolver o paciente do HC para a atenção primária, por exemplo, e aí o paciente está acompanhando sem indicação no serviço terciário.* (Daniela, geriatra)

Esse tipo de limitação não é exclusivo desta UBS, e parte do que se atribui como baixa autonomia do idoso, ou um exercício da autonomia que limita a ação dos serviços, precisa ser reinterpretado à luz do que está sendo efetivamente oferecido ao idoso como possibilidade de cuidado. De fato, excesso de demanda, numerosa população vinculada às equipes, demora para agendamentos, burocracia, falta de informatização e poucos recursos são problemas apontados em geral por usuários e profissionais da saúde^3^^,^^27^^,^^28^^,^^29^.

## CONSIDERAÇÕES FINAIS

A generalização de achados de uma pesquisa qualitativa deve ser examinada com bastante atenção, pois a singularização da situação examinada, necessária ao aprofundamento interpretativo, compete com a extensividade das categorias com que se constrói o estudo. O que se espera é que a fecundidade hermenêutica do quadro compreensivo produzido possa ser útil a outros contextos comparáveis.

Os resultados aqui apresentados serão mais facilmente transferíveis ao contexto de idosos vivendo em grandes centros urbanos, de sociedades ocidentais industrializadas, em segmentos sociais de renda média e média baixa, com acesso a serviços de atenção primária à saúde e algum grau de suporte social via políticas públicas. Isto não significa, porém, que o estudo não tenha relevância para outros contextos. O modo como foram configuradas aqui as relações entre autonomia e processos saúde-doença-cuidado de idosos pode ajudar a reconfigurar a compreensão dos desafios da atenção à saúde do idoso em contextos diversos. Pode ser particularmente útil, nesse sentido, a crítica a leituras individualizantes do valor da autonomia e, ao contrário, a ênfase no seu poder analisador sobre práticas sociais e programáticas relacionadas, e não apenas para idosos, mas também para outras situações em que a questão da autonomia seja relevante para o cuidado, como no caso de pessoas com deficiência ou transtornos mentais.

Conforme o exposto, no contexto da atenção primária, as dificuldades encontradas no cuidado do idoso perpassam a autonomia plasmada com as vulnerabilidades às quais cada idoso está exposto em determinado momento. Olhar para as necessidades de cuidado do idoso por uma ótica relacional e processual de seus limites é um caminho potencial para que se veja a autonomia não como uma finalidade em si mesma, muito menos como um obstáculo ou uma resistência que precise ser superada para proteger os idosos dos riscos trazidos pelo envelhecimento, mas sim como um marcador do *quê* e *como* precisa ser transformado (individual, social e programaticamente) para se cuidar efetivamente, isto é, para mobilizar recursos técnicos que potencializem sucessos práticos no que se refere à saúde.

A maior ou menor autonomia que reclama um ou outro indivíduo, neste ou naquele aspecto do seu cotidiano, sinaliza, na verdade, regiões de vulnerabilidade, áreas relacionais (interpessoais, sociais, programáticas) que requerem atenção especial para que êxito técnico e sucesso prático possam de fato interagir de forma construtiva e adequada aos projetos de felicidade dos idosos.

Ao ser construída e compreendida apenas nos contextos concretos de interação cotidiana dos idosos, a autonomia vai ganhando diferentes feições em decorrência das distintas situações da prática, o que impõe pensá-la não como um atributo individual (que o idoso *ainda tem* ou que *já perdeu*), mas como um marcador de características relacionais que reclamam estratégias técnicas e horizontes prático-morais plurais e flexíveis, sempre desde um horizonte ético de respeito ao outro e responsabilidade em relação a ele.
